# A metasynthesis of qualitative studies regarding opinions and perceptions about barriers and determinants of health services’ accessibility in economic migrants

**DOI:** 10.1186/1472-6963-12-461

**Published:** 2012-12-17

**Authors:** Andrés A Agudelo-Suárez, Diana Gil-González, Carmen Vives-Cases, John G Love, Peter Wimpenny, Elena Ronda-Pérez

**Affiliations:** 1 Faculty of Dentistry, University of Antioquia, Calle 64 N° 52-59, Medellín, Antioquia, Colombia; 2 Public Health Research Group, University of Alicante, Campus de San Vicente del Raspeig s/n, Alicante, 03690, Spain; 3 Observatory of Health Policies and Health (OPPS), University of Alicante, Campus de San Vicente del Raspeig s/n, Alicante, 03690, Spain; 4 CIBER of Epidemiology and Public Health (CIBERESP), Madrid, Spain; 5 School of Applied Social Studies. Faculty of Health & Social Care Aberdeen, The Robert Gordon University, Aberdeen, United Kingdom; 6 Joanna Briggs Collaborating Centre. Faculty of Health and Social Care. School of Nursing and Midwifery, The Robert Gordon University, Aberdeen, United Kingdom

**Keywords:** Health care, Health services accessibility, Emigrants and immigrants, Systematic review, Qualitative research, Metasynthesis

## Abstract

**Background:**

Access to health services is an important health determinant. New research in health equity is required, especially amongst economic migrants from developing countries. Studies conducted on the use of health services by migrant populations highlight existing gaps in understanding which factors affect access to these services from a qualitative perspective. We aim to describe the views of the migrants regarding barriers and determinants of access to health services in the international literature (1997–2011).

**Methods:**

A systematic review was conducted for Qualitative research papers (English/Spanish) published in 13 electronic databases. A selection of articles that accomplished the inclusion criteria and a quality evaluation of the studies were carried out. The findings of the selected studies were synthesised by means of metasynthesis using different analysis categories according to Andersen’s conceptual framework of access and use of health services and by incorporating other emergent categories.

**Results:**

We located 3,025 titles, 36 studies achieved the inclusion criteria. After quality evaluation, 28 articles were definitively synthesised. 12 studies (46.2%) were carried out in the U.S and 11 studies (42.3%) dealt with primary care services. The participating population varied depending mainly on type of host country. Barriers were described, such as the lack of communication between health services providers and migrants, due to idiomatic difficulties and cultural differences. Other barriers were linked to the economic system, the health service characteristics and the legislation in each country. This situation has consequences for the lack of health control by migrants and their social vulnerability.

**Conclusions:**

Economic migrants faced individual and structural barriers to the health services in host countries, especially those with undocumented situation and those experimented idiomatic difficulties. Strategies to improve the structures of health systems and social policies are needed.

## Background

Health has been recognised as a fundamental human right, regardless of sex, political affiliation, social class or ethnicity, as well as the right to minimum conditions of wellbeing, including the provision of medical care and public services for all people [1]. International organisations as United Nations highlight how important it is to ensure these rights, and call for such inequalities to be addressed by identifying their determinants [2]. Reforms in the political and social systems of many countries have also had an impact both on how health systems are organised and on health service user profiles and access [3].

Access to health services is considered a determinant of health inequalities [4]. In terms of the provision of such services, Tudor-Hart’s inverse care law [5] identified that population groups with the highest health needs -the most deprived and vulnerable groups in society- tended to receive the least health care provision, whilst those with the least health need -the most affluent and advantaged societal groups- received the most health care. In relation to access to, and use of, health services, conceptual frameworks have been developed, such as that of Andersen [6-8], which stress that access to health services should be analysed from the perspective of health policy objectives, the characteristics of the health system, and the results obtained: input (factors affecting service use) and output (health status and health behaviours). Tanahashi in 1978 [9] proposed a schematic model of health service coverage and utilization, and outline several aspects related to the utilization of health services in terms of the interaction between specific aspects of service provision (service capacity) and the characteristics of the target population (service target).

It is well known that poor health is disproportionately experienced by those on the margins of society and living in disadvantaged socio-economic condition and migrants are represented amongst these groups [10]. Although international migrations are highly heterogeneous, they occur mainly for economic reasons [11]. Economic migrants are defined as people of working age (16–65 years), born outside the country in which they are employed or are residing -either permanently or for an extended period of time. They come from developing countries (Latin-America, Eastern Europe, Africa and Asia) [12]. Research has emphasized that before arrival, migrant populations are characterised by a good state of health but this is often eroded by the migratory process itself and by the living and working conditions experienced in the host country [13].

Studies have been conducted on the use of health services by migrant populations and ethnic minority groups and highlight existing gaps in understanding which factors affect access to these services, by focusing upon structural and individual factors [14-16]. Such studies have sought to formulate propositions that will help to improve communication between service provider and user, thereby enabling the development of effective social and health policies. Of increasing importance is the use of qualitative research in public health studies, in order to capture the subjective experience and individuals’ perceptions of their own health, in order to understand how determinants influence the relationship between migrants and health, and to identify causes for existing inequalities between migrant and non-migrant groups [17]. Accordingly, the present review and analysis of the scientific literature has included methodologies to evaluate qualitative studies and draw together the findings, through metasynthesis [18]. As such, this systematic review seeks to describe economic migrants’ views of barriers and determinants to health services’ accessibility by identifying, evaluating and synthesising qualitative research that relates to the experience of access to health services by economic migrant populations within the period 1997–2011.

## Methods

An international systematic review was carried out of to identify all qualitative studies whose primary focus was to illuminate the barriers and determinants of health services accessibility amongst economic migrants. The search covered the time period January 1997 to November 2011. In the choice of this period were taken into account factors: 1) some countries since the nineteenth century had greatest migratory tradition, like the U.S., Canada, New Zealand or Argentina (which are called classic immigration countries). However, in the territory the influx of European immigrants is much later [19]. For example, between 1998 and 2007, the migrant worker population in European OECD (Organisation for Economic Co-operation and Development) countries increased significantly, from 3.5 to almost 6 million workers [20,21]; 2) it is important to consider population movement linked to the massive social upheavals and significant world economic conditions and migration created by world events beginning in 1997, such as European, Eastern Block changes, the opening up of China’s boundaries and changes to migration policies in a number of developed countries [22]; 3) after an initial search in databases, a list of potential papers for the systematic review were found in the decade of 90’s. For that means, the research team decided to establish the point cut for beginning the systematic search in 1997 based on current scientific criteria.

### Inclusion and exclusion criteria

The review sought to identify all qualitative research studies related to health services accessibility by economic migrants (based upon phenomenology, ethnography, grounded theory, ethnomethodology, phenomenography and critical, interpretative or feminist analyses). These had to be focused on at least one of three aspects: barriers, conditioning and facilitator factors, and the impact of health services as a determinant of migrants’ health. The review considered studies that included economic migrants. We excluded studies with populations of migrants coming from developed countries, tourists, students and relatives of those economic migrants who constitute a separate category (family class/reunification) with different health statuses and behaviours.

### Search strategy and data extraction

The initial search included Medline and CINAHL to identify text words contained in the title and the abstract as well as classify the appropriate descriptor/MeSH terms to be used. The next step used identified keywords and index terms in 13 electronic databases in Health and Social Sciences (see Table 1).

**Table 1:** Summary of the search strategy used for the systematic review

**1.**	**Type of literature**	**Source**
a	Published material	· Medline,
		· The Excerpta Medica Database –EMBASE-,
		· The Cumulative Index to Nursing and Allied Health –CINAHL-,
		· Sociological Abstracts,
		· Scopus,
		· Lilacs,
		· ISI Web of Knowledge -Web of Science, Current Contents and ISI Proceedings- and
		· Applied Social Sciences Index and Abstracts –ASSIA
B	Grey Literature	· The Centre for Health Care Strategies,
		· OpenSIGLE,
		· The International Centre for Migration and Health,
		· The UC atlas of Global inequality and
		· Google Scholar.
**2.**	**Search Terms**	· Free terms: Delivery of health care, Health care, Public health, Healthcare disparities, Health Services Accessibility, Immigrant, Migrant (Demographic, Economic, Socioeconomic, Cultural boundaries).
		· MeSH terms (U.S National Library of Medicine 2011): Immigrant and [delivery of health care; health care -public health-; healthcare disparities; Health services accessibility].
		· For the other databases, the strategy was adapted accordingly to the specific thesaurus and free terms.

**Figure 1:**
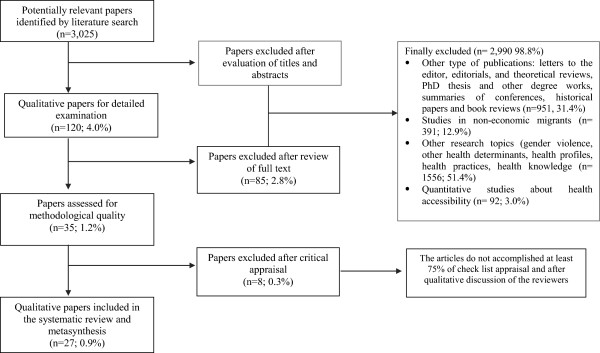
Exclusion and inclusion scheme.

We located 3,025 potentially relevant papers and 120 (4.0%) of these were selected on title and abstract by the lead reviewer. This process was checked by a second reviewer. The full text of these papers was then reviewed and subsequently, after a first reading and application of inclusion/exclusion criteria, 35 (1.2%) studies were selected for critical appraisal. The decision of excluding 85 papers is based on different characteristics related mainly with the topic of the studies that are not related with barriers to accessibility to health services, for example: studies conducted in no-economic migrants, studies based in health providers’ perspectives [23], studies in other topics such as: gender violence [24], other health determinants [25,26], health practices [27], culture and health [28], health knowledge [29] and recommendations for community-based strategies [30]. Two reviewers of the research team appraised papers independently. The process of complete data extraction is explained in Figure 1. 

### Critical appraisal and studies’ analysis

Appraisal of included papers for methodological quality was undertaken using a critical appraisal checklist and data extraction form for interpretive and critical research [31] (Table 2). Any disagreements that arose between reviewers were resolved through discussion with at least one other member of the research team and we discarded 8 papers [32-39]. The 27 (0.9%) selected articles were those satisfying at least 75% of the checklist of the critical appraisal [40-66]. 

**Table 2:** **Critical appraisal checklist for qualitative research**[31]

**Reviewer**	**Date**
**Author**	**Year**
**Record Number**	
For each statement is evaluated (Yes-No-Unclear)	1. Is there congruity between the stated philosophical perspective and the research methodology?
	2. Is there congruity between the research methodology and the research question or objectives?
	3. Is there congruity between the research methodology and the methods used to collect data?
	4. Is there congruity between the research methodology and the representation and analysis of data?
	5. Is there congruity between the research methodology and the interpretation of results?
	6. Is there a statement locating the researcher culturally or theoretically?
	7. Is the influence of the researcher on the research, and vice- versa, addressed?
	8. Are participants, and their voices, adequately represented?
	9. Is the research ethical according to current criteria or, for recent studies, and is there evidence of ethical approval by an appropriate body?
	10. Do the conclusions drawn in the research report flow from the analysis, or interpretation, of the data?
	11. Overall appraisal: Include___ Exclude___ Seek further info.___ Comments (Including reasons for exclusion)

The review followed the principles of meta-synthesis [67-70]. This process involved the aggregation of findings and categories to generate a set of synthesised statements that represented aggregation through categorisation of findings related in meaning. Initially, manual analysis was performed to identify categories related with accessibility to health services through several readings of the papers. Subsequently, data were entered into the JBI QARI software, which is designed to manage the synthesis of textual data as part of a systematic review of evidence [71]. Several triangulation strategies were employed to improve the quality of the data and conclusions; by source (the different migrant collectives studied and different health systems/welfare systems), different methodologies in the papers analysed, different conceptual frameworks and discussion by the research team. We identified from the papers the following information: First author (year), setting, data collection, participants, data analysis, services referred in the study, barriers to and determinants of access to health services for the migrant population, inputs and outputs in the access to health services and facilitative conditions and strategies for overcoming barriers. The extracted information of the studies are presented in different analysis categories following the Andersen’s conceptual framework of access to, and use of, health services, the categories related with other studies conducted in migrants and new categories emerging from the different discourses extracted from the papers. All the advances of the process of this systematic review were discussed in academic meetings for the research team in other to guarantee the quality of the information and this paper has been elaborated considering the PRISMA guidelines for reporting systematic reviews [72].

## Results

Table 3 and Table 4 show the general characteristics of the studies considered in the metasynthesis. 12 studies (44.4%) were carried out in the U.S [40-51] and 6 (22.2%) in Canada [54-59]. 12 studies (44.4%) dealt with primary care services [40-45,52,56,59,63-65]. The participating population varied depending mainly on type of host country. The studies carried out in the USA focused mainly on the Latino population [40-43,45,48,49,51], two with Asian migrants [44,47] and Muslim population [46] with one specific study focusing on the indigenous population from one state in Mexico [50]. In case of other countries, some studies carried out in Australia, Canada and New Zealand are focused on Asian population [53-57,64]. Studies in Israel focused on Russian, Ethiopian and Arab migrants [60-62]. One study in Australia deals with a population from Brazil [52], and one study in Canada also included Romanian migrants [57]. One study carried out in Spain was explicit in mentioning the economic migrant population from five regions: the Maghreb, Sub-Saharan Africa, Asia, Latin America and Eastern Europe [65]; the other Spanish study was focused on Ecuadorian population living in Barcelona [66]. 

**Table 3:** Characteristics of the studies included in the review

**First author (year)**	**Data collection**	**Participants**	**Data analysis**	**Services referred in the study**
Boyer LE (2001) [40]	Interviews in participant’s homes	20 Hispanic women 18/65 years of age	Content analysis/Analysis for themes, patterns and categories	Primary health care
Buki LP (2004) [41]	Focus groups (5 FG)	Latina women (n = 58) from Mexico (n = 13), Puerto Rico (n = 10), Cuba (n = 12), El Salvador (n = 12) and South America (n = 11).	Grounded theory/Analysis for themes, patterns and categories	Primary health care
Pinzon-Perez H (2005) [42]	Semi-structured interviews	Migrant Latina women (n = 51)	Phenomenological approach	Primary health care
Goodman MJ (2006) [43]	Focus group (9 FG)	Latino patients (n = 70)	Content analysis	Primary health care
Gany FM (2006) [44]	Focus groups (13 FG)	Caribbean (2FG), Cantonese/Chinese (2FG), Mandarin/Chinese (1FG), Haitian community (2FG), Korean Community (2FG), Latino community (4FG). Total 108 (44 M, 64 F)	Inductive analysis techniques: Analysis for themes, patterns and categories.	Primary health care
Natale-Pereira A (2008) [45]	Focus groups (5 FG)	Latino staff members from 5 community based organizations in New Jersey (8 M, 28 F)	Grounded theory	Primary health care
Simpson JL (2008) [46]	In depth interviews (n = 7)	Muslim Arabic Women (n = 7)	Phenomenological approach	Primary health care
Wu MC (2009) [47]	Focus Groups (4FG)	Korean Migrants (Community Women n = 15)	Content Analysis	Mental Health Services
Cristancho S (2004) [48]	Focus groups: "focused small groups discussions" (n = 19)	Hispanic migrants 181 from Mexico, Guatemala, El Salvador, Colombia, Cuba and Puerto Rico	Analysis for themes, patterns and categories	Non-specified
Garces I (2006) [49]	Focus group (8 FG, n = 54)	Migrant Latina women (n = 54)	Identification of categories in Theoretical model (PEN-3)	Non-specified
McGuire S (2006) [50]	In depth semi structured interviews (n = 22)	Indigenous Oaxacan women (n = 22)	Dimensional coding/Theoretical and operational memos/Explanatory matrix.	Non-specified
Harari N (2008) [51]	Semi-structured interviews (n = 50)	Latino migrant (32 F, 18 M)	Content analysis/Analysis for themes and categories	Non-specified

Setting: U.S.

**Table 4:** Characteristics of the studies included in the review

**First author (year)**	**Setting**	**Data collection**	**Participants**	**Data analysis**	**Services referred in the study**
Leite da Silva A (2004) [52]	Australia	Participant observation, in depth individual interviews (n = 33), focus group (8 FG)	Brazilian migrant women (n = 33)	Open, selective and axial coding (by using a computer program for the analysis of qualitative data QSR Nvivo)	Primary health care, public and private
Blignaut I (2008) [53]	Australia	In depth interviews (n = 33)	Community members (9 F, 4 M), Chinese mental health patients (8 F, 1 M)	Utilization of codes into categories and subcategories Nvivo.	Mental Health Services
Johnson JL (2004) [54]	Canada	Open-ended interviews (n = 50). Focus group (6 FG- 30 participants).	South Asian India, Pakistan, Bangladesh, Fiji and East Africa Women (n = 80) from different religions Sikh, Hindu, Muslim, Christian.	Ethnographic techniques/Analysis for themes, patterns and categories	Non-specified
Whitley R (2006) [55]	Canada	In depth interviews (n = 15)	West Indian migrants (11 F, 4 M)	Ethnographic techniques.	Mental Health Services
Wang L (2008) [56]	Canada	Focus groups (2 FG), field observations	Chinese migrants (n = 15)	Descriptive analysis	Primary health care
Asanin J (2008) [57]	Canada	Focus groups (14 FG)	Migrants from Pakistan, India, China, Romanian others 53	Grounded theory	Non-specified
Dean JA (2010) [58]	Canada	In depth interviews (n = 23)	Migrants from Africa, Asia, Eastern Europe, Latin America/Carribbean, Europe	Inductive analysis	Non-specified
Poureslami I (2011) [59]	Canada	Focus Groups	Latino, Chinese, Iranian and Punjabi communities	Descriptive analysis	Primary health care
Remennick L (1998) [60]	Israel	Qualitative interviews	Russian Migrants	Content analysis	Non specified
Elnekave E (2004) [61]	Israel	Qualitative phase: Participant Observation, long and short/semi-structured interviews (80 F), focus group	Arab Israeli women	Analysis for themes, patterns and categories	Non-specified
Shtarkshal RA (2009) [62]	Israel	Semi-structured interviews	Ethiopian Migrants (14)	Analysis through socio-ecological model	Non-specified
Suurmond J (2011) [63]	Netherlands	Semi-structured individual and group interviews with 22 participants	7 non-Dutch origins (Chile, China, Turkey, Dominic Republic, Portugal, Italia, Surinam	Deductive analysis from a framework method	Primary and specialized
Zhang W (2008) [64]	New Zealand	Face to face interviews (n = 21)	Chinese migrants (11 F, 10 M)	Analysis for themes, patterns and categories	Dental health services Primary health care
Ramos M. (2001) [65]	Spain	Focus groups (3 FG), Nominal groups (NG = 3), Partially structured interviews (n = 14)	Economic migrants	Analysis for themes, patterns and categories	Primary health care
Terrasa-Nuñez R (2010) [66]	Spain	Semi-structured interviews (n = 18)	Ecuadorian migrants (8 F, 10 M)	Inductive analysis	Non-specified

Setting: Rest of countries.

**Figure 2:**
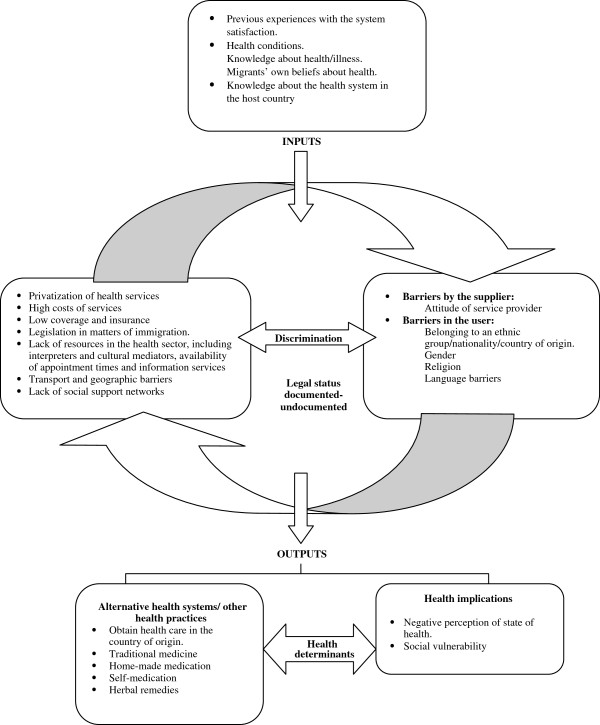
Barriers and determinants in health care access from the migrants’ perspective*. * Summary of the results of the articles included in the metasynthesis.

Figure 2 shows the summary of studies showing the identified barriers from the point of view of economic migrant and the elements that determine this access. In general terms, factors such as the knowledge of the health system in the host country, the health status or Migrants’ own beliefs/knowledge about health are previous characteristics (inputs) and could constitute social determinants, previous to the utilization of health services in economic migrants. Secondly, migrants identified barriers that could be classified in those related with the structure or the social security/health system in the host country such as: economic barriers (cost of services), health services and insurance coverage, privatization of the services, and other related with the attitude and communicative abilities to the provider (health personnel) and barriers that belong to the migrant condition (language skills, cultural competence, religion). Discrimination appeared as an important social determinant of health services accessibility related with individual and structural characteristics. Lastly, the low/lack of utilization of health services could affect negatively the health profile of the economic migrants and caused the searching of alternative ways to improve their health (alternative medicine, self-medication).

The results and implications from the different studies are deeply presented below.

### Barriers to and determinants of access to health services for the migrant population

First of all, the studies identify barriers based on factors operating at three different levels: firstly, the structural/political level (i.e. cultural, policy and resource considerations that determine the scale and configuration of services); secondly, the institutional factors (i.e. factors relating to service organisation and delivery) and the individual factors (i.e. the characteristics of migrants themselves and service providers, including socio-demographic characteristics, knowledge, communication skills and motivation).

In terms of the structural/political level, studies identified a range of obstacles, including the lack of coverage in health and social security programme, health insurance, or in some cases being unable to access private insurance due to a lack of resources and health service costs [40,44,46,48,49,52,57,64].

Also, elements were identified relating to the impact of social and economic policies on the privatisation of health services [52]; the lack of clarity in immigration legislation that hinders health care is also considered important [65].

Finally, the documented status (legal or illegal) of migrants in the host country affects their use of health services, restricts service availability and system coverage and insurance, and access to private health insurance [45,48,50,55,56,66].

With respect to the organisational factors, the characteristics of health care services, in terms of availability of human resources [57], interpreters and cultural mediators [47,48,62], opening hours and availability of medical and dental services [40], the lack of information about different programmes and services [51,63,66], and the perceived quality of the services provided [42,54] were all determinants of use health services amongst migrants taking part in the various studies analysed. In addition, geographical barriers and transport difficulties due to the location of health services with regard to their places of residence and work were also identified [47,48,57,64,66].

Individual factors constituted the third main type of obstacle that acted to exclude migrants from health services. Thus, a lack of social support networks left participants in a vulnerable and isolated situation in their communities and in the host country, with consequent impact upon health service use [51,52]. Elsewhere, studies reported barriers resulting from cultural aspects to do with belonging to a particular ethnic group [40,41,54,56,57,59,62], or for having a particular nationality or country of origin, religion [46] or sex [61]. In some cases, participants expressed feelings of inferiority in the health system of the host country or of not feeling welcome [51].

Further, many of the studies also identified language barriers [42,45,48-53,56,57,61,64] that hinder communication between service provider and patient, which led to feelings of insecurity among migrants [66].

Finally, discrimination in the health services from being an migrant, or for one’s sexual condition [43], by institutions themselves and resulting from health care workers’ attitudes [42,48,52-55,60,62-64], are a determinant that influences health practices and the utilisation of health services, and lead to feelings of mistrust by migrants towards organisations and health professionals [40,52].

### Inputs and outputs in the access to health services

Inputs in access to health services refer to the information and characteristics that have an influence, as external determinants, on the barriers experienced by migrants. The qualitative studies identified elements such as prior attitudes and opinions regarding health services [41,57], beliefs, knowledge, values and attitudes about health [44,45,57,58,63,64], and the priority of health over other aspects relating to social and living conditions, or over legalising their current situation [40,45,50,65]. The degree of knowledge about the health system in the host country is also an influence [46,47,53,63,64,66].

Outputs refer to the implications of the determinants and barriers to migrants accessing health services, which affect their negative perception or lack of control over their health [49,52] and their feelings of social vulnerability [61]. The migrant population uses “alternative support ways” when they do not receive care from the health services. These support ways include using traditional Eastern medicine [55,56] home-made remedies [45,49] and self-medication [51] as well as obtaining health care in their countries of origin [51]. In this last aspect, migrants make a comparison between health systems, establishing that the treatment they receive from health professionals is better in their country of origin, and that the costs and the technology are higher in the host country [48,52,58,64].

### Facilitative conditions

Some facilitative conditions and strategies “to overcome barriers” were found in the studies analysed. For example, recommendations are made by the participants in the studies, who recognise the role that support institutions and social networks play [47,51,66] in improving certain social and health conditions, as well as the contribution made by community organisations and health teams, which work in different neighbourhoods delivering education and health-promotion campaigns [45,47]. They also recognise family support as fundamental for making decisions and receiving health care [49,62]. Where language difficulties exist, relatives act as translators [51,62]. Preferences were also identified for professionals that speak the same language as the migrants [41]. Other recommendations for the health systems and health services are regarding strategies involving flexible payment for services provided [51]. Some studies identify positive aspects in health plans and services -such as for the obstetric population- [51] and the technical quality of health professionals [49,54]. One study revealed that migrants improved their health situation because of improvement of living standards in comparison to the origin country [58].

## Discussion

With the results of this review it was possible to consider the obstacles that economic migrants face when accessing health services, by using the participants’ discourse to define a typology of barriers that encompass structural/political, organisational and individual factors [6-8]. Thus, macro-level social and economic policies, including immigration policies and the privatisation of health services, influenced the availability and configuration of health services for migrants [9]. Also, the organisation and delivery of health services at a local level, including service opening hours, the geographical siting of services and the availability of translators influenced health service use amongst migrants [15]. In addition, a range of individual factors, residing in the migrants themselves (e.g. socio-demographic characteristics, knowledge and motivation) and in the service providers (e.g. staff attitudes) informed health service use amongst migrants [15].

The studies included were carried out in seven countries (Australia, Canada, USA, Israel, New Zealand, Netherlands and Spain), the first four of which have a longstanding tradition of immigration dating back to the 1980s and the phenomenon is much more recent in New Zealand and Spain [73-76]. This is significant because access barriers must be analysed within the context of the host countries and their reforms concerning to health systems.

According to the overall performance report by the World Health Organization (2000) that compare the health system of the countries [77], Spain was in 7th place in health system performance, Netherlands was 17th, Israel 28th, Canada 30th, Australia 32nd, USA 37th and New Zealand 41st. This includes factors such as general level of health, response capacity and how financial contributions are spread. For economic migrants, inequalities in access to health services would be explained not only by their socioeconomic level [78] but also by factors relating to the countries’ overall policies and strategies [77].

Many of the studies were carried out in the USA, where the health system is run by various private entities, and operates different health programmes that vary by State. In this regard, the US literature reports that 15% of the population have no insurance [79] and structural barriers exist that lead to inequalities in care among vulnerable collectives, such as the lack of a regular health care resource, a lack of funding, and geographic barriers [14,16,80].

The Canadian health system, meanwhile, is universal and is both publicly and privately financed [81]. However, access barriers exist due to the length of time one must spend as a resident in the country in order to access health care services, the complexity of the system [82] and the high cost of private insurance services [83], and the reduction of economic benefits [23]. Australia has a universal public insurance system, but the studies show difficulties in terms of access to programmes and services for the undocumented (illegal) population [84], and barriers relating to service cost [36]. New Zealand has a mixed system, with public insurance funded through taxation, which does not cover some services, such as dental care -only for children and under 18 s- [85]; some migrants access private services to request a quicker and more timely service in comparison with public services, and for some medical and dental procedures and treatment, with the inconvenience of high costs [86]. In Israel, the insurance system is universal and works with various government institutions [87-89], and the impact of insurance policies (National Health Insurance Law) on minority groups in Israeli society, such as Arabs [61], Ethiopians [62] and Russians [60], has been discussed. Healthcare in the Netherlands is financed by a dual system and several reforms have been carried out [77]. Lastly, in case of Spain, the insurance system is universal and the guarantee of health care in similar conditions to the autochthonous people, nevertheless could exist administrative bureaucratic difficulties affecting the use of health services for instance a lack of knowledge of the Spanish system organization and the different requirements for accessing [90,91].

In general terms, the barriers experienced by the migrant population described in the various studies are in relation to the insurance system of the host country, with differences depending on each country’s context. In addition, it is important to take in account the models of social welfare and the capacity of the states to provide welfare services and benefits in social security, health care, housing, education and social services for all people and in case of the present review, for the migrants [92,93].

The barriers expressed by the participants with regard to language difficulties and belonging to different ethnic groups related to the migrants’ values system and beliefs within a particular culture [15], as well as the culturalisation processes of groups in the host societies, which hinder proper integration and thus affect how health services are used [94]. In some cases, this means feeling discriminated against by different institutions [95], feeling unaccepted and misunderstood or perceiving a certain attitude towards them by health professionals [15]. Legal status is also a factor contributing to greater vulnerability, due to a precarious social and employment situation, despite the fact that, in some contexts, health care is universal [96,97]. This suggests that health services should adapt to the social and economic context, offering medical attention according to individuals’ particular needs and cultural aspects.

Barriers to accessing health services have implications in both the subjective perception of a poor state of health [98] and in the use of alternative health systems to resolve a health need. This situation depends on cultural aspects and on social inequalities between groups, leading to increased social vulnerability [2].

One of the strengths of this review (despite the fact that it deals with heterogeneous studies from different countries) is that this is the first time that findings describing determinants and barriers affecting access to health services have been synthesised in this way, analysing the discourses and perceptions of those directly involved in the process. There is increasing interest in qualitative research in the field of public health [99]. Awareness among the international scientific community should therefore be raised with regard to the importance of recognising participants’ discourse as a research objective. There is still certain timidity in qualitative studies, given that they mention sample size and the impossibility of generalising results as limiting factors. Qualitative research does not aim to be statistically representative, but rather as an approach that has proved to be useful in filling “gaps” that are not resolved by quantitative research, in particular examining issues such as understanding and motivation.

This systematic review has limitations that should be highlighted. Although studies were selected by an exhaustive search of scientific and grey literature databases, there may be unpublished reports. The systematic search and the initial process of extracted data was in charge of the review leader, however, the process was supervised for a second reviewer in order to evaluate the accordance of the selected articles for further analysis. We have used an instrument to evaluate the quality of qualitative research, and although this has proven to be effective in other studies, it is important to recognise that evaluating the literature of qualitative studies depends on the subjective evaluation of the researchers, although throughout the process the consensus and agreement among the research group was guaranteed. More information is needed on inequalities access to health care, considering aspects such as gender and social class and further research is needed into strategies that help migrants to minimise the negative effects of access barriers. It seems important to research the impact of health reforms on vulnerable collectives.

Furthermore, it is important to recognise the difficulty in selecting studies dealing with economic migrant populations. This metasynthesis focuses on populations that are migrants for economic and work-related reasons, and the literature is based mainly on belonging to a minority ethnic group in the destination country being a proxy of migrant status, and although they are similar, they are not equivalent [100,101] given that, in the current context, the second or third generations of migrants tend to acquire nationality of the destination country. There are also migrants for political or social reasons, who may have differing characteristics to the economic-type.

The results of the studies analysed are circumscribed by a number of factors including, study design, the characteristics of the populations interviewed and the data-gathering techniques used. These include heterogeneous and variable data analysed with regard to the populations chosen [40]; limiting findings to a particular geographic, social and political context [40,50,57]; difficulty in generalising results by the sample used in the studies -not random-, and by participant selection [41-46,48,49,51,53,55,56,62,64]; and by the characteristics of the qualitative techniques -interview and focus group, among others- [65]; cultural background and language difficulties of participants, which can hinder field work [42,46].

## Conclusion

Dealing with social inequalities in the migrant population requires greater knowledge of the social determinants and the relationships that occur within social inequalities, in order to provide health-equality strategies in plans and policies, in terms of health as a universal right [102], a better handling of health care resources, strengthening public and universal health care systems and improving living conditions for vulnerable collectives.

## Competing interests

The authors declare that they have no competing interests.

## Authors’ contributions

All the authors contribute with the data analysis, the written of the manuscript and the approbation of the final version to be submitted to the journal.

## Pre-publication history

The pre-publication history for this paper can be accessed here:

http://www.biomedcentral.com/1472-6963/12/461/prepub
